# Efficacy and safety of combined TACE-HAIC and targeted immunotherapy in primary liver cancer with portal vein tumor thrombus

**DOI:** 10.3389/fonc.2026.1811934

**Published:** 2026-06-15

**Authors:** Shaoqin Yang, Shuangshuang Zhang, Yuming Gu

**Affiliations:** 1 Xuzhou Medical University, Xuzhou, Jiangsu, China; 2 Interventional Radiology Department, The Affiliated Hospital of Xuzhou Medical University, Xuzhou, Jiangsu, China

**Keywords:** hepatic arterial infusion chemotherapy, portal vein tumor thrombus, primary liver carcinoma, propensity score matching, transarterial chemoembolization

## Abstract

**Objective:**

This study aims to assess the efficacy and safety of combining transarterial chemoembolization and hepatic arterial infusion chemotherapy (TACE-HAIC) with targeted immunotherapy in patients with primary hepatocellular carcinoma (HCC) complicated by portal vein tumor thrombus (PVTT).

**Methods:**

We retrospectively analyzed clinical data from 114 patients with HCC and PVTT treated with either a quadruple-therapy group (TACE-HAIC plus targeted immunotherapy) or a triple-therapy group (TACE plus targeted immunotherapy) at the Affiliated Hospital of Xuzhou Medical University from January 2019 to September 2023. Propensity score matching was applied to these 114 patients. We compared outcomes such as overall survival (OS), progression-free survival (PFS), intrahepatic progression-free survival (IHPFS), PVTT progression-free survival (TTPFS), objective response rate (ORR), disease control rate (DCR), and the incidence of adverse events between the groups.

**Results:**

The quadruple-therapy group exhibited a markedly superior ORR of portal vein tumor thrombus relative to the triple-therapy group (P<0.05). However, no significant differences were found between the groups in OS, PFS, IHPFS, or TTPFS (P > 0.05).

**Conclusion:**

The combination of TACE-HAIC with targeted immunotherapy (the quadruple-therapy group) is an effective treatment strategy for PVTT, significantly inducing regression of intraportal tumor thrombi and achieving a higher objective response rate.

## 1 Introduction

Combined local and systemic therapy has become the principal approach for treating hepatocellular carcinoma (HCC) with portal vein tumor thrombus (PVTT) (1).Transarterial chemoembolization (TACE) is a well-established interventional therapy, supported by a long history and substantial evidence from evidence-based medicine. Its combined application with tyrosine kinase inhibitors and immune checkpoint inhibitors demonstrates a significant therapeutic effect in patients with advanced liver cancer (2, 3). Hepatic artery infusion chemotherapy (HAIC) elevates the local concentration of chemotherapeutic agents while diminishing adverse events such as ectopic embolism. The Japanese Society of Hepatology (JSH) explicitly recommends HAIC as a first-line treatment for patients with HCC-PVTT (4). In recent years, multiple phase III clinical trials have confirmed that oxaliplatin combined with fluorouracil and calcium folinate chemotherapy (FOLFOX)-HAIC treatment significantly prolongs the survival time of patients with unresectable liver cancer compared with TACE treatment (5, 6). However, research on patients with vascular invasion remains limited. This paper aims to evaluate the efficacy and safety of TACE-HAIC combined with targeted immunotherapy in patients with HCC-PVTT, thereby providing a stronger foundation for selecting clinical interventional therapies for these patients.

## 2 Materials and methods

### 2.1 General information

**Figure 1:**
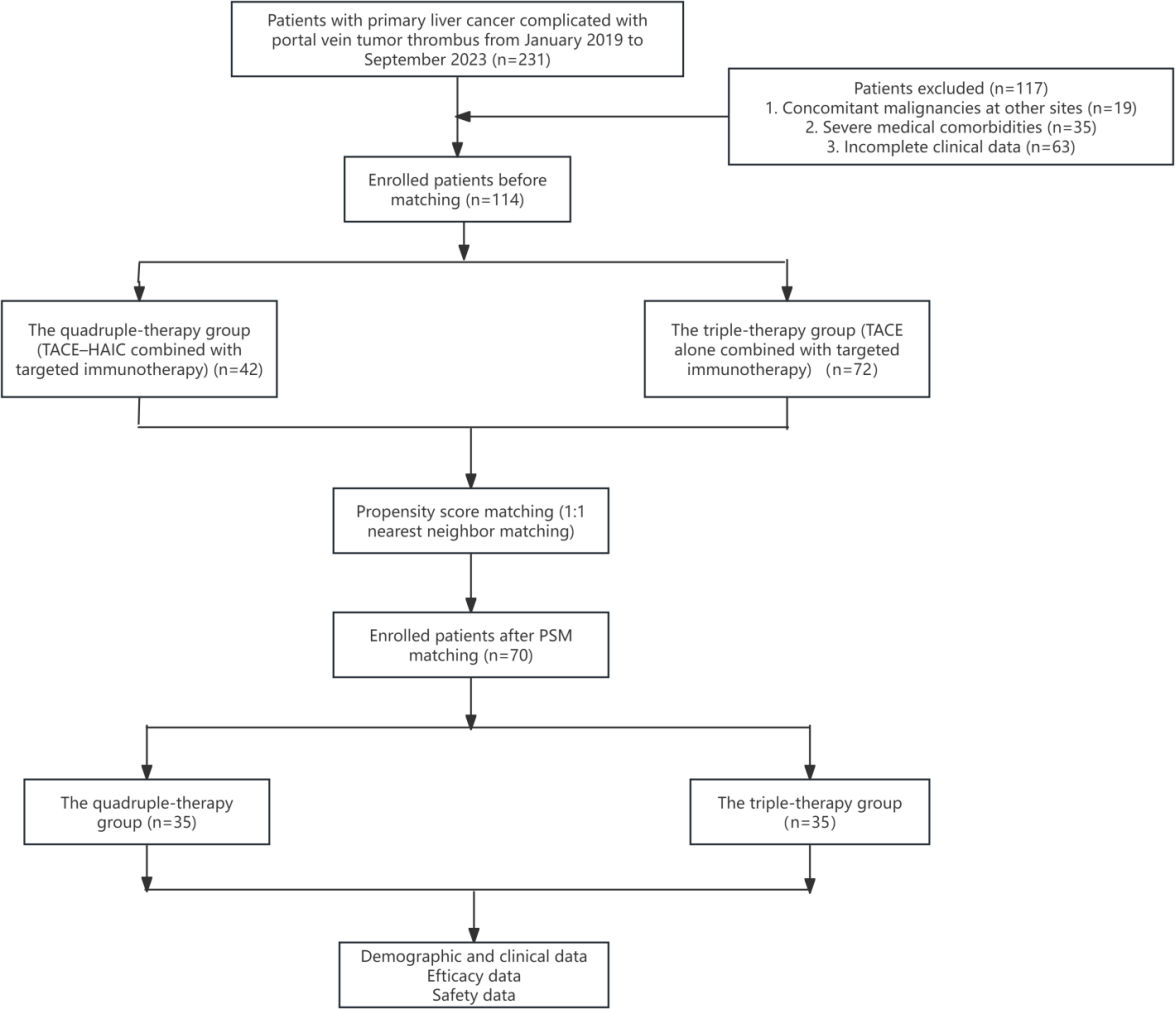
Flowchart.

Clinical data from HCC-PVTT patients treated with either combined TACE-HAIC therapy or TACE alone at the Affiliated Hospital of Xuzhou Medical University between January 2019 and September 2023 were collected. Inclusion criteria included: ① HCC diagnosis via imaging or pathology, with portal vein tumor thrombus confirmed by CT/MRI; ② At least one session of TACE-HAIC/TACE treatment received; ③ At least one measurable hepatic lesion per mRECIST criteria; ④ Available imaging monitoring data, such as enhanced CT, MRI, or contrast-enhanced ultrasound; ⑤ sSufficient hematological monitoring data, including complete blood count, liver function, and tumor markers. Exclusion criteria were: ① Concomitant malignancies at other sites; ② Severe medical comorbidities, such as significant cardiac, pulmonary, renal, or coagulation dysfunction.③The clinical data were incomplete. 114 patients were enrolled and assigned to two treatment groups: 42 patients in the quadruple-therapy group (TACE–HAIC combined with targeted immunotherapy) and 72 patients in the triple-therapy group (TACE alone combined with targeted immunotherapy). The study flowchart is presented in **Figure 1**.

This study was reviewed and approved by the Medical Ethics Committee of the Affiliated Hospital of Xuzhou Medical University (Approval No.: XYFY2025-KL533-01).

### 2.2 Treatment methods

Both groups underwent TACE treatment. Following successful arterial puncture, the catheter was placed in the celiac trunk or common hepatic artery for angiography. This procedure confirmed the tumor’s location, size, number, feeding arteries, and the presence of any hepatic artery-hepatic vein or portal vein fistula. The tumor-feeding artery was then super-selectively embolized. Initially, appropriate chemotherapy drugs were administered, followed by embolization of intrahepatic lesions using a mixed emulsion of chemotherapy drugs and super-liquefied iodized oil. Finally, gelatin sponge or drug-loaded microspheres were used to embolize the tumor-feeding artery until its blood flow was completely halted. The treatment process was tailored based on the tumor’s location, size, and number. In the TACE-HAIC group, the catheter was retained after successful TACE treatment, and upon safe return to the ward, a continuous infusion of oxaliplatin, calcium folinate, and fluorouracil was administered. Oxaliplatin (85 mg/m²) in 100 mL of 0.9% saline was infused continuously for 3 hours, levoleucovorin (200 mg/m²) in 50 mL of 5% dextrose for 6 hours, and 5-fluorouracil (2400 mg/m²) in 1000 mL of 0.9% saline for 46 hours. In contrast, in the TACE group, the catheter was removed immediately after treatment, and the puncture site was compressed and bandaged. Patients in both groups received lenvatinib and immune checkpoint inhibitors (primarily camrelizumab and sintilimab) beginning 1 week after the initial intervention. Abdominal contrast-enhanced CT and MRI were performed at regular intervals to evaluate the lesions.

### 2.3 Efficacy evaluation

Reexamine abdominal contrast-enhanced CT, MRI, or color Doppler ultrasound contrast every 4 weeks, and evaluate the tumor treatment response according to the mRECIST criteria. Overall survival (OS) was defined as the period from HCC diagnosis to death from any cause. Progression-free survival (PFS) was measured from HCC diagnosis to the first radiological progression or death. Intrahepatic progression-free survival (IHPFS) and tumor thrombus progression-free survival (TTPFS) were defined from treatment initiation to the first radiological progression of intrahepatic lesions and portal vein tumor thrombus (PVTT) or death due to HCC, respectively. For PVTT evaluation, complete response (CR) was marked by the complete disappearance of PVTT enhancement in the arterial phase. Partial response (PR) was noted with any downstaging in Vp’s classification, while progressive disease (PD) was indicated by any upstaging. Otherwise, it was considered stable disease (SD) (7–9). The objective response rate (ORR) was the percentage of patients achieving CR or PR, and the disease control rate (DCR) included those with CR, PR, or SD.

### 2.4 Safety evaluation

Safety evaluation encompasses the incidence and severity of adverse events during treatment, graded and assessed using the Common Terminology Criteria for Adverse Events (CT-CAE) version 5.0. Patients’ general conditions, including nausea, vomiting, abdominal pain, and diarrhea, are recorded alongside blood routine, liver function indicators, and thyroid function changes. All adverse events are systematically documented at each follow-up visit until 4 weeks post-treatment.

### 2.5 Statistical analysis

A logistic regression model estimated the propensity scores for matching between two patient groups. To minimize confounding effects, covariates such as gender, age, ECOG score, HBV infection, ascites, portal hypertension, maximum tumor diameter, number of lesions, extrahepatic metastasis, Child-Pugh classification, AFP value, and cancer embolus type were included. A 1:1 matching was conducted with a caliper value of 0.02. Categorical variables were analyzed using the χ² test or Fisher’s exact test, while the t-test or Mann-Whitney U test was applied for inter-group comparisons. Survival curves were generated using the Kaplan-Meier method, with the Log-rank test employed for group comparisons. Univariate and binary logistic regression analyses were performed on variables influencing tumor progression. A p-value of less than 0.05 was considered statistically significant. Statistical analysis were performed using SPSS27.0 and R 4.5.0.

## 3 Results

Clinical data from 114 patients were gathered. Imaging criteria for the diagnosis of portal hypertension included a main portal vein diameter of ≥ 1.3 cm and a splenic vein diameter of ≥ 1.0 cm (as measured on contrast-enhanced CT or MRI). To minimize potential selection bias and confounding effects of treatment, a propensity score matching analysis (PSM) was conducted. The patients’ baseline data are presented in **Table 1**.

**Table 1:** Baseline data of patients in the two groups.

Clinical data	Before PSM			After PSM		
	The quadruple-therapy	The triple-therapy	P value	The quadruple-therapy	The triple-therapy	P value
Gender						
Male	40	63	0.210	34	33	1.000
Female	2	9		1	2	
Age(years)						
<65	34	55	0.570	28	29	0.759
≥65	8	17		7	6	
HBV infection						
Yes	33	59	0.660	29	31	0.495
No	9	13		6	4	
ECOG score						
0	15	27	0.849	13	15	0.626
1	27	45		22	20	
Ascites						
Yes	15	23	0.680	13	10	0.445
No	27	49		22	25	
Portal hypertension						
Yes	14	33	0.191	11	8	0.083
No	28	39		14	27	
Maximum tumor diameter						
≤7cm	24	44	0.677	22	19	0.467
>7cm	18	28		13	16	
Number of lesions						
≤3	16	42	0.037	15	13	0.626
>3	26	30		20	22	
Extrahepatic metastasis						
Yes	1	3	1.000	1	1	1.000
No	41	69		34	34	
AFP value(μg/L)						
≤400	20	36	0.806	17	21	0.337
>400	22	36		18	14	
Child-Pugh classification						
A	18	44	0.059	17	21	0.337
B	24	28		18	14	
PVTT grade						
Vp2	18	38	0.493	14	15	0.834
Vp3	21	26		18	17	
Vp4	3	8		3	3	
Targeted-immunotherapy cycles	20	13	0.307	21	12	0.299

**Figure 2:**
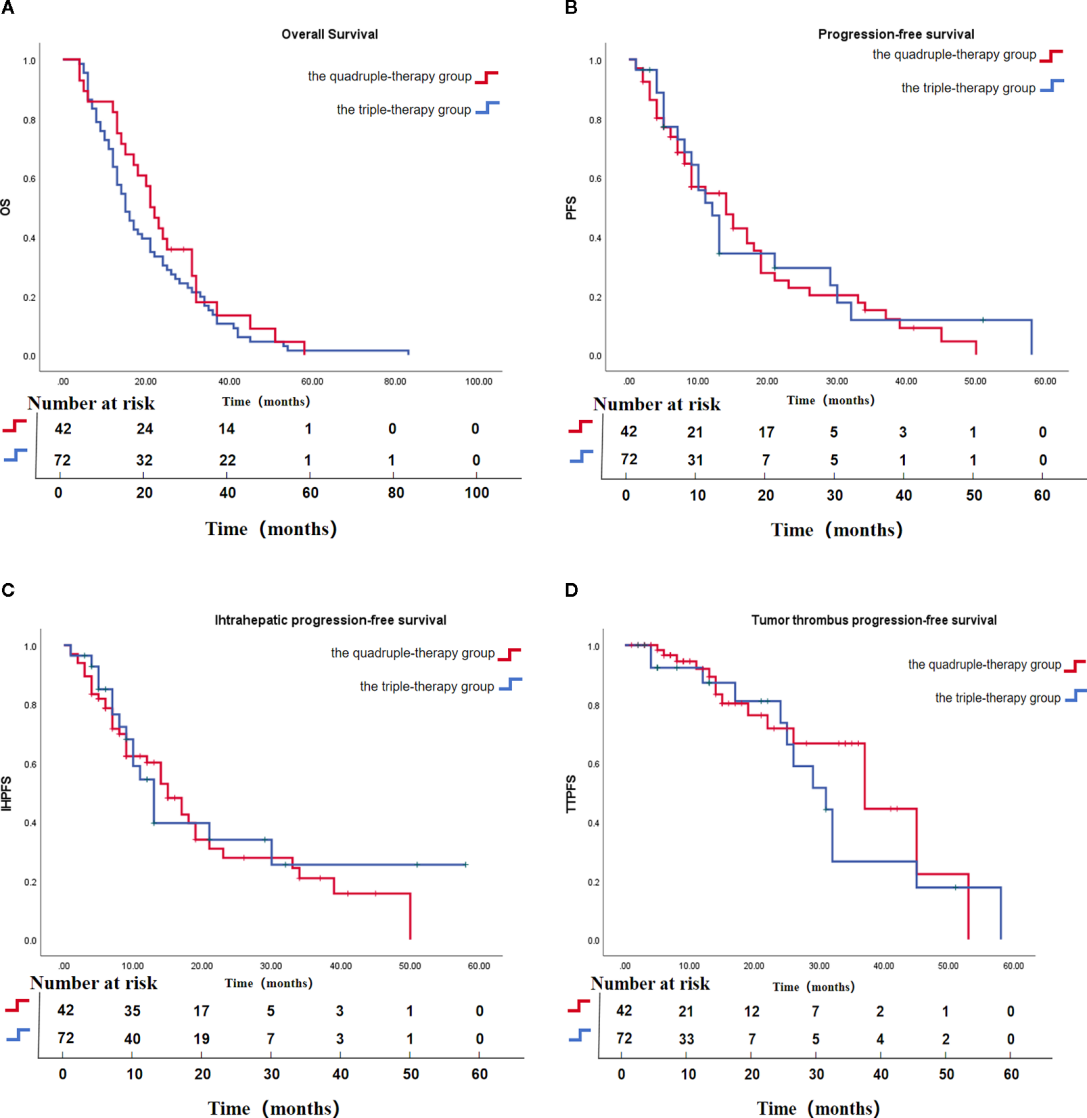
Comparison of survival curves between the quadruple-therapy group and the triple-therapy group before PSM. **(A)** OS; **(B)** PFS; **(C)** TTPFS; **(D)** IHPFS.

**Figure 3:**
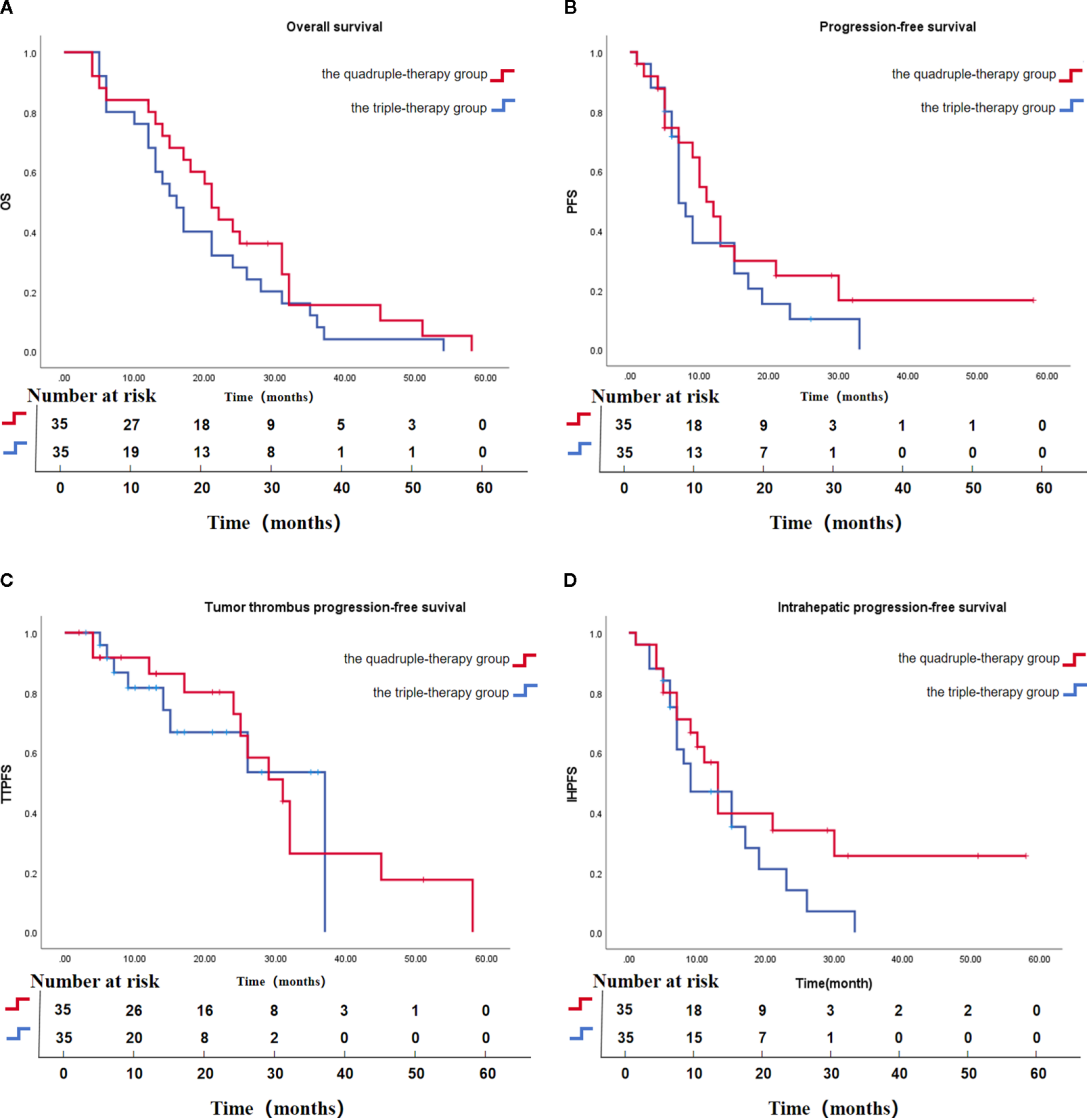
Comparison of survival curves between the quadruple-therapy group and the triple-therapy group after PSM. **(A)** OS; **(B)** PFS; **(C)** TTPFS; **(D)** IHPFS.

The median follow-up period for patients was 17 months. Before PSM, the median OS, PFS, TTPFS, and IHPFS for the quadruple-therapy group compared to the triple-therapy group were:21 months vs 15 months (HR:0.784,95% CI:0.497-1.238,P = 0.289), 10 months vs 8 months (HR:0.918,95% CI:0.542-1.556,P = 0.352),15 months vs 13 months (HR:0.873,95% CI:0.639-1.992,P = 0.296),and 10 months vs 9 months (HR:0.900,95% CI:0.502-1.615,P = 0.320). After PSM, the median OS, PFS, TTPFS, and IHPFS for the quadruple-therapy group compared to the triple-therapy group were: 21 months vs 16 months (HR:0.797,95% CI:0.389-1.460,P = 0.224),11 months vs 8 months (HR:0.840,95% CI:0.441-1.600,P = 0.310),16 months vs 13 months (HR:0.801,95% CI:0.417-1.238,P = 0.081),and 11 months vs 8 months (HR:0.727,95% CI:0.366-1.444,P = 0.171).None of these differences reached statistical significance (**Figures 2**, **3**).

The analysis of tumor treatment response indicated no statistically significant differences in ORR and DCR for intrahepatic lesions between the two patient groups. However, a statistically significant difference was observed in the ORR of portal vein tumor thrombus (**Tables 2**, **3**). To identify independent factors influencing ORR, a univariate analysis was initially performed, revealing that the treatment method was a predictive factor for ORR. Subsequently, variables potentially affecting the progression of portal vein tumor thrombus were included in a multivariate analysis, which confirmed that the treatment method remained an independent predictive factor (**Table 4**).

**Table 2:** Tumor response of portal vein tumor thrombus.

Tumor response	Before PSM			After PSM		
	The quadruple-therapy	The triple-therapy	P value	The quadruple-therapy	The triple-therapy	P value
CR	7(16.7%)	5(6.9%)	0.044	7(20.0%)	3(8.6%)	0.027
PR	12(28.6%)	12(16.7%)		11(31.4%)	4(11.4%)	
SD	10(23.8%)	25(34.7%)		9(25.7%)	16(45.7%)	
PD	13(30.9%)	30(41.7%)		8(22.9%)	12(34.3%)	
ORR	45.3%	23.6%	0.017	51.4%	20.0%	0.006
DCR	69.1%	58.3%	0.255	77.1%	65.7%	0.290

**Table 3:** Tumor response of intra-hepatic lesion.

Tumor response	Before PSM			After PSM		
	The quadruple-therapy	The triple-therapy	P value	The quadruple-therapy	The triple-therapy	P value
CR	3 (7.1%)	5 (6.9%)	0.077	2 (5.7%)	1 (2.9%)	0.188
PR	12 (28.6%)	14 (19.4%)		12 (34.3%)	11 (31.4%)	
SD	17 (40.5%)	22 (30.6%)		14 (40.0%)	9 (25.7%)	
PD	10 (23.8%)	31 (43.1%)		7 (20.0%)	14 (40.0%)	
ORR	35.7%	26.3%	0.294	40.0%	34.3%	0.621
DCR	76.2%	56.9%	0.039	80.0%	60.0%	0.069

**Table 4:** Univariate and multivariate analyses of factors influencing the objective response rate in portal vein tumor thrombus.

Variables	Before PSM				After PSM			
	Univariate analyses	Multivariate analyses			Univariate analyses	Multivariate analyses		
		OR	95%CI	P value		OR	95%CI	P value
Gender	0.602				1.000			
Age	0.890				0.705			
HBV infection	0.836				0.234			
ECOG score	0.033	0.338	0.123-0.929	0.036	0.630	0.514	0.116-2.280	0.381
Ascites	0.397				0.157			
Portal hypertension	0.527				0.198			
Maximum tumor diameter	0.127	0.473	0.158-1.413	0.180	0.470	1.003	0.229-4.391	0.997
Number of lesions	0.686	0.967	0.349-2.680	0.948	0.630	1.296	0.306-5.498	0.725
Extrahepatic metastasis	0.891	1.088	0.073-16.179	0.951	0.530	0.000	0.000	0.999
AFP value (μg/L)	0.751	1.216	0.460-3.211	0.846	0.869	0.468	0.109-2.006	0.370
Child-Pugh classification	0.824	1.105	0.403-3.035	0.693	0.869	0.679	0.153-3.011	0.610
PVTT grade	0.214			0.451	0.130			0.172
local and systemic therapy (TACE-HAIC/TACE)	0.022	3.263	1.146-9.291	0.027	0.018	6.658	1.474-30.061	0.014

Throughout the treatment process, neither group experienced grade 3 or higher adverse reactions, and no deaths from toxic reactions were observed. Patients in the TACE-HAIC group, however, experienced nausea and vomiting more frequently than those in the sole TACE group, though these symptoms were alleviated with active treatment. This increased incidence may be attributed to the additional chemotherapy drugs (**Table 5**). In the quadruple therapy group, a total of 16 patients experienced treatment interruption due to treatment-related adverse events, compared with 21 patients in the triple therapy group(38.1% vs 29.2%,P=0,326).Adverse events associated with quadruple therapy are manageable and reversible, and did not lead to treatment discontinuation due to toxicity.

**Table 5:** Treatment-related adverse reactions.

Adverse reactions	Before PSM			After PSM		
	The quadruple-therapy	The triple-therapy	P value	The quadruple-therapy	The triple-therapy	P value
Leukopenia	13	28	0.720	12	10	0.569
Thrombocytopenia	14	34	0.893	12	10	0.569
Anemia	10	21	0.713	8	11	0.382
Nausea and vomiting	14	18	0.033	12	5	0.037
Abdominal pain	13	30	0.931	11	9	0.564
Diarrhea	6	15	0.890	3	8	0.171
Elevated aminotransferase	8	20	0.867	5	7	0.722
Elevated bilirubin	16	32	0.443	14	13	0.777
Decreased albumin	7	17	0.939	6	4	0.725
Renal toxicity	8	15	0.547	7	8	0.758
Hand - foot syndrome	7	13	0.566	5	7	0.508

## 4 Discussion

Portal vein tumor thrombus (PVTT) is the most prevalent form of vascular invasion in hepatocellular carcinoma (HCC), with an incidence ranging from 44.0% to 62.2%. This condition progresses rapidly and is associated with a poor prognosis. Untreated patients have a median survival time of only 2.7 to 4 months, and the 1-year survival rate is below 15% (10, 11). Currently, transarterial chemoembolization (TACE) combined with systemic therapy is the preferred treatment for HCC-PVTT patients. However, TACE presents several unresolved challenges: ① PVTT restricts the portal vein’s blood supply, and TACE further compromises hepatic blood flow, potentially worsening liver function and causing severe complications such as elevated bilirubin, liver failure, ascites, gastrointestinal bleeding, and hepatic encephalopathy;②TACE-induced hypoxia can trigger the release of vascular growth factors, promoting tumor microvessel formation and leading to tumor recurrence and progression (12). ③Patients with PVTT often develop arterioportal shunts (APFs), heightening the risk of ectopic embolism. He et al. (13), and Zhang et al. (14) reported that, in HCC patients with PVTT, HAIC produces rapid tumor shrinkage, reduces hepatic burden, and suppresses thrombus progression, while also improving survival and tumor response. Xu (15) further proposed that HAIC can reach PVTT via hepatic portal venous shunting and exert direct antitumor effects on the thrombi.

This study revealed that for patients with HCC-PVTT, the combination of TACE-HAIC and targeted immunotherapy significantly promoted the regression of portal vein tumor thrombus (PVTT). The objective response rate was notably higher than that in the group receiving TACE alone. and the safety profile was manageable. Traditional TACE primarily targets the arterial blood supply of tumors, but its effectiveness is limited for the core of PVTT, which is mainly fed by the portal vein (16). In contrast, HAIC continuously delivers high-concentration chemotherapy drugs through an indwelling catheter, allowing these drugs to diffuse into tumor tissue along the concentration gradient, thereby effectively targeting PVTT. Multiple studies and meta-analyses have also confirmed that the quadruple regimen of TACE/HAIC combined with targeted therapy and immunotherapy is significantly superior to the TACE combined with targeted therapy and immunotherapy regimen in terms of ORR, which is consistent with the findings of this study (17, 18). Furthermore, our study also found that among the 42 patients in the quadruple therapy group, 3 patients (7.1%) achieved tumor downstaging and subsequently underwent hepatectomy. All three patients had an overall survival of more than 24 months. In contrast, although several patients in the triple therapy group achieved tumor downstaging, none received surgical resection or conversion therapy. The quadruple therapy regimen can improve tumor response and disease control rates, thereby providing more opportunities for conversion therapy and the potential for curative resection in HCC patients with PVTT.

However, the study also found that despite the higher remission rate of PVTT, this did not translate into significant improvements in overall survival and progression-free survival. This may be due to several factors:① survival benefits usually require a sufficiently long follow-up period to be fully manifested. A meta-analysis demonstrated that the OS superiority of TACE/HAIC plus targeted immunotherapy began to expand continuously after 18 months of follow-up (19). The median follow-up time in our study was 17 months, which may not have reached the time threshold for significant separation of survival curves. ②All patients received subsequent targeted combined immunotherapy. Although no significant difference was observed in the number of subsequent treatment cycles between the two groups, the specific therapeutic regimens were not entirely uniform, which might also contribute to the heterogeneity of survival outcomes. ③ The small sample size may have resulted in low statistical power, making it difficult to detect potentially clinically significant survival differences between the two groups.

Limitations of this study include: ① Being a retrospective, single-center study with a small sample size, it inherently suffers from selection bias, even with the use of propensity score matching (PSM), resulting in uncontrollable confounding bias. ② While all patients eventually underwent targeted combination immunotherapy, the specific treatment regimens varied. Due to the limited sample size, subgroup analysis was not feasible. Future research is necessary to examine different systemic treatment regimens. ③ According to the mRECIST criteria, PVTT is classified as a non-target lesion. The guidelines define CR for non-target lesions as the complete disappearance of arterial phase enhancement, without requiring complete radiological resolution of the lesion. Therefore, in this study, the absence of arterial phase enhancement was adopted as the criterion for determining CR of PVTT, consistent with the unified standard. However, this evaluation method has certain limitations. The disappearance of arterial phase enhancement only indicates the necrosis of tumor activity, whereas non-enhancing necrotic tumor thrombus may still persist within the portal vein lumen, failing to accurately reflect the anatomical resolution of PVTT. Future studies are warranted to develop a more rigorous and tailored efficacy evaluation system specifically for PVTT.

Our findings indicate that TACE-HAIC combined with targeted immunotherapy is an effective treatment for PVTT. While its effect on overall survival remains uncertain, the quadruple-therapy clearly improves ORR of portal vein tumor thrombus, rapidly reduces tumor burden, and facilitates downstaging. Therefore, it represents a viable option for translational or neoadjuvant therapy in intermediate- and advanced-stage hepatocellular carcinoma. These conclusions are generally consistent with prior reports (20–23). Future large-scale, prospective randomized controlled trials are essential to further validate the survival benefits of this combination regimen and to identify the optimal patient population that could benefit most, considering factors such as tumor thrombus classification and liver function status.

## Data Availability

The original contributions presented in the study are included in the article/supplementary material. Further inquiries can be directed to the corresponding author.
